# Anti-oxidant anti-inflammatory and antibacterial tannin-crosslinked citrate-based mussel-inspired bioadhesives facilitate scarless wound healing

**DOI:** 10.1016/j.bioactmat.2022.05.017

**Published:** 2022-05-21

**Authors:** Keke Wu, Meimei Fu, Yitao Zhao, Ethan Gerhard, Yue Li, Jian Yang, Jinshan Guo

**Affiliations:** a Department of Histology and Embryology, NMPA Key Laboratory for Safety Evaluation of Cosmetics, School of Basic Medical Sciences, Guangdong Provincial Key Laboratory of Bone and Joint Degeneration Diseases, The Third Affiliated Hospital of Southern Medical University, Southern Medical University, Guangzhou, China; b Department of Biomedical Engineering, Pennsylvania State University, University Park, PA, 16802, USA

**Keywords:** Scarless wound healing, Tannic acid, Hydrogen bond crosslinking, Anti-oxidant, Phased angiogenesis

## Abstract

The revolutionary role of tissue adhesives in wound closure, tissue sealing, and bleeding control necessitates the development of multifunctional materials capable of effective and scarless healing. In contrast to the use of traditionally utilized toxic oxidative crosslinking initiators (exemplified by sodium periodate and silver nitrate), herein, the natural polyphenolic compound tannic acid (TA) was used to achieve near instantaneous (<25s), hydrogen bond mediated gelation of citrate-based mussel-inspired bioadhesives combining anti-oxidant, anti-inflammatory, and antimicrobial activities (3A-TCMBAs). The resulting materials were self-healing and possessed low swelling ratios (<60%) as well as considerable mechanical strength (up to ∼1.0 MPa), elasticity (elongation ∼2700%), and adhesion (up to 40 kPa). The 3A-TCMBAs showed strong *in vitro* and *in vivo* anti-oxidant ability, favorable cytocompatibility and cell migration, as well as photothermal antimicrobial activity against both *Staphylococcus aureus* and *Escherichia coli* (>90% bacterial death upon near-infrared (NIR) irradiation). *In vivo* evaluation in both an infected full-thickness skin wound model and a rat skin incision model demonstrated that 3A-TCMBAs + NIR treatment could promote wound closure and collagen deposition and improve the collagen I/III ratio on wound sites while simultaneously inhibiting the expression of pro-inflammatory cytokines. Further, phased angiogenesis was observed via promotion in the early wound closure phases followed by inhibition and triggering of degradation & remodeling of the extracellular matrix (ECM) in the late stage (supported by phased CD31 (platelet endothelial cell adhesion molecule-1) PDGF (platelet-derived growth factor) and VEGF (vascular endothelial growth factor) expression as well as elevated matrix metalloprotein-9 (MMP-9) expression on day 21), resulting in scarless wound healing. The significant convergence of material and bioactive properties elucidated above warrant further exploration of 3A-TCMBAs as a significant, new class of bioadhesive.

## Highlights

• Citrate-based mussel-inspired bioadhesive prepolymers were crosslinked with tannic acid via hydrogen bonding (3A-TCMBAs).
• 3A-TCMBAs showed good tissue adhesiveness, self-healing and elastic properties.
• 3A-TCMBAs exhibited photothermal antibacterial, antioxidant and anti-inflammatory efficiency.
• 3A-TCMBAs could promote scarless wound healing by enabling phased angiogenesis.

## Graphical abstract

**Figure:**
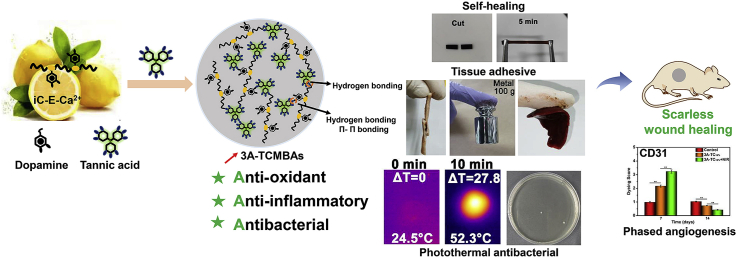

## 1 Introduction

The largest organ in the human body, skin functions as a critical barrier against external invasion; however, damage via surgery, burns, and other accidents mediates adverse health events including bleeding and microbial infection. Infection and other pro-inflammatory factors left unchecked lead to chronic inflammatory reactions and significantly delayed wound healing [[1], [2], [3]]. During the inflammatory phase, immune cells secrete pro-inflammatory cytokines, inducing the production of reactive oxygen species (ROS), such as hydrogen peroxide (H_2_O_2_), hydroxyl radical (OH·) and superoxide anion radical (O_2_˙^−^), by resident inflammatory cells [[4], [5], [6]]. Excessive accumulation of ROS in infected wounds can overwhelm the native antioxidant defense system, strengthen the inflammatory reaction, and thus delay wound healing [7,8] and cause scar formation [[9], [10], [11], [12]], resulting in skin dysfunction and adverse cosmetic appearance [13]. ROS-scavenging biomaterials including polyphenols [14,15], nanozymes [16,17], and others, exhibit enhanced therapeutic effects via augmentation of malfunctioning or depleted native antioxidant mechanisms, indicating their utility in the resolution of delayed wound closure and scarless wound healing. The development of a multifunctional therapeutic material system combining antimicrobial capacity, ROS scavenging, and promotion of skin regeneration is thus highly desirable.

Wound dressings are widely used clinically due to ease of application and significant therapeutic effects; however, traditional wound dressings (e.g., cotton, bandages and gauze) lack functionality (antioxidant and antibacterial properties) and are unable to provide a moist environment [[18], [19], [20], [21]]. Tissue adhesives or adhesive hydrogels are an effective alternative to both traditional surgical sutures and wound dressings, able to provide a moist environment for wound healing [22]. As a subclass, mussel-inspired bioadhesives incorporating l-DOPA (L-3,4-dihydroxyphenylalanine) or dopamine have been the subject of intensive research as a result of strong wet tissue adhesion strengths caused by the formation of covalent bonds between catechol groups and amino/thiol groups on the tissue surface [[23], [24], [25], [26]]. Moreover, the anti-oxidant capacity of dopamine can effectively relieve oxidative stress and improve the speed of wound healing [27,28]. In our previous work, a series of injectable citrate-based mussel-inspired bioadhesives (iCMBAs) have been developed via the facile one-step polycondensation of citric acid (CA), polyol, dopamine, and other functional moieties [23,24,29]; however, significant toxicity concerns have arisen due to the use of harsh oxidants, such as sodium periodate (PI), silver nitrate (SN), and iron (III) chloride (FeCl_3_), as crosslinking initiators. Significantly, the use of such strong oxidants also abolished the anti-oxidant potential of the incorporated catechol moieties, motivating the search for a new crosslinking strategy minimizing the toxicity and maintaining the anti-oxidant capacity of mussel-inspired bioadhesives.

**Scheme 1:**
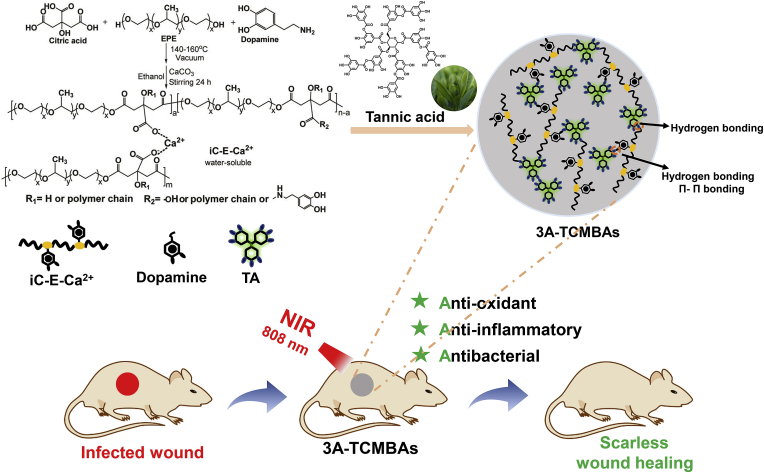
Synthesis mechanism of 3A-TCMBAs and application for scarless wound healing.

Tannic acid (TA), a typical plant-derived polyphenol, has become increasingly attractive as an alternative to dopamine, with strong adhesion and excellent anti-oxidant and antibacterial properties [[30], [31], [32], [33], [34]]. Additionally, TA is a low-cost material approved by the U.S. Food and Drug Administration (FDA) as a safe and biocompatible component for medical device development, enhancing its clinical potential [35]. Leveraging its large number of carbonyl and phenolic functional groups, TA is able to bond to various polymers such as poly(ethylene glycol) (PEG) [36], chitosan (CS) [37], silk fibroin (SF) [38], and thioctic acid [14] through multiple reaction pathways, including electrostatic interactions, hydrogen bonding, hydrophobic interactions and covalent bonding [39,40]. Herein, we translated the impressive bonding ability of TA into development of a series of self-healing, low swelling, and tissue-adhesive citric acid-based bioadhesives via simple physical mixing of TA with previously developed iCMBA prepolymers. The resulting materials exhibited biocompatibility and excellent antibacterial ability (Scheme 1). Avoiding traditional strong oxidant crosslink initiators and supplementing iCMBA with the strong anti-oxidant and antimicrobial activities of TA, simultaneous anti-oxidant, anti-inflammatory and antimicrobial activities were achieved. The physical and mechanical properties, degradation profiles, tissue adhesion strengths, anti-oxidant properties, biocompatibility and NIR photothermal antimicrobial activity of these anti-oxidant, anti-inflammatory and antibacterial tannin-crosslinked citrate-based mussel-inspired bioadhesives (3A-TCMBAs) were thoroughly investigated both *in vitro* and *in vivo*. The wound healing capability of 3A-TCMBAs was also studied in an infected full-thickness skin wound model and a rat skin incision model. Overall, 3A-TCMBAs serve as a versatile multifunctional therapeutic platform for wound closure and healing.

## 2 Materials and methods

### 2.1 Materials

Tannic acid (TA, Mw = 1701.2 Da) and 2, 2-diphenyl-1-picrylhydrazyl (DPPH, 96%) were purchased from Macklin Reagent. Poly(ethylene glycol)-block-poly(propyleneglycol)-*block*-poly(ethylene glycol) (PEG-PPG-PEG, EPE, Pluronic®L-31, M_w_ = 1100 Da), citric acid (CA), dopamine (DA) and calcium carbonate (CaCO_3_) were purchased from Sigma-Aldrich. All other chemical reagents were of analytical grade and were used without further purification.

### 2.2 Synthesis of iC-E-Ca^2+^ prepolymers

EPE containing and calcium carbonate treated injectable citrate-based mussel-inspired bioadhesive (iC-E-Ca^2+^) prepolymers were synthesized via the one pot polycondensation of citric acid (CA), EPE and dopamine (DA) according to previous literature [41], followed by treatment with excess CaCO_3_ to ionize pendent carboxyl groups [26]. Briefly, CA, EPE and DA at a molar ratio of 1.2: 1.0: 0.3 were charged to a one-necked round-bottom flask and heated to 160 °C under stirring until the reactants were melted. After that, the temperature was reduced to 140 °C, and the reaction was continued until the stir bar stopped turning at 60 rpm. Then, the reaction mixture was dissolved in ethanol, and excess CaCO_3_ (2–10 folds to CA) was added and the mixture was stirred for 12 h. Finally, the mixture was dialyzed in deionized (DI) water, filtered and lyophilized, and freeze-dried iC-E-Ca^2+^ prepolymer was obtained for further study.

### 2.3 Preparation of 3A-TCMBAs

The iC-E-Ca^2+^ prepolymer was dissolved in DI water to form a 33 wt% solution, and then 5, 10, 15, or 20 wt% of TA solution was used as a crosslinking initiator. The anti-oxidant, anti-inflammatory and antibacterial tannin-crosslinked citrate-based mussel-inspired bioadhesives (3A-TCMBAs) were fabricated by simply mixing the iC-E-Ca^2+^ prepolymer solution and TA solution with a volume ratio of 2: 1. All adhesives were allowed to cure for 24 h and then freeze-dried for further characterization. The 3A-TCMBA samples crosslinked by TA solutions with different concentrations were named 3A-TC_5%_, 3A-TC_10%_, 3A-TC_15%_ and 3A-TC_20%_.

### 2.4 Gel time, sol content, swelling ratio, and degradation study of 3A-TCMBAs

The gel times of different samples were measured via a tilting test. For each sample, testing was conducted three times and the results were averaged. The sol content of 3A-TCMBAs were measured as described previously [41] and then calculated using the following equation:

$$\text{Sol content }\left(\text{\%}\right)=\frac{W_{i}-W_{d}}{W_{i}}\times 100\%$$

where *W*_*i*_ and *W*_*d*_ represent the initial mass of dry hydrogel sample and freeze-dried sample after leaching with 1, 4-dioxane for 48 h, respectively.

The swelling ratios of the 3A-TCMBAs were measured by gravimetric analysis. The freshly prepared wet sample was weighed (*W*_*0*_), and then separately immersed in PBS solution (pH 7.4) for a specific time at 37 °C until an equilibrium weight was reached. The surface water was removed by filter paper before the swollen weight (*Wt*) of the hydrogel was measured at a desired interval. The swelling ratios of 3A-TCMBAs were calculated using the following equation [42,43]:

$$\text{Swelling ratio }\left(\text{\%}\right)=\frac{W_{t}}{W_{0}}\times 100\%$$

The degradation performance of 3A-TCMBAs was investigated in phosphate buffered saline (PBS, 01 M, pH 7.4) at 37 °C. 3A-TCMBA hydrogels were lyophilized and the dry weights were recorded (*W*_*0*_). Then, the samples were immersed in 10 mL of PBS and incubated at 37 °C. At predetermined time points, the samples were washed with DI water and lyophilized to record the residual mass (*W*_*t*_). The degradation percentage was calculated using the following equation [44]:

$$\text{Degradation }\left(\text{\%}\right)=\frac{W_{0}-W_{t}}{W_{0}}\times 100\%$$

### 2.5 Adhesion strength

The adhesive strengths of 3A-TCMBAs were measured using the lap shear strength test according to a modified ASTM D1002-05 method. In order to mimic adhesion to soft tissue, 3A-TCMBAs were applied to the surface of porcine skin tissue with a bonding area of 10 mm × 10 mm. The adhered tissue strips were compressed with a 100 g weight for 30 min and then were placed in a high humidity chamber for 2 h prior to testing. The lap shear strength of bonded tissue strips was subsequently measured using an Instron 34TM-10 fitted with a 10 N load cell at a rate of 1.3 mm/min. The adhesive strengths of 3A-TCMBAs to different substrates including iron, rubber, glass, and plastic were also conducted.

### 2.6 Rheological and self-healing properties of 3A-TCMBAs

Rheological testing of 3A-TCMBAs was carried out using a TA Instruments, DHR-2. Time sweeps with 1% constant strain and a constant frequency of 10 rad/s at 37 °C were used to evaluate the stiffness of the 3A-TCMBAs. The polymer mixture was placed between two 20 mm parallel plates, and the periphery was sealed with silicone oil to prevent the evaporation of water. Alternating small strain (1%) and large strain (100%) scans were also conducted for four cycles to study the self-healing property of 3A-TCMBAs.

### 2.7 Mechanical properties of 3A-TCMBAs

The tensile mechanical profiles of dried and swollen 3A-TCMBAs were measured employing an Instron materials test system (Instron 34TM-10) equipped with a 500 N load cell. Dried 3A-TCMBAs films were cut into rectangles (25 mm length × 6 mm width × 1.5 mm thickness) and stretched to failure at a strain rate of 500 mm/min. The Young's modulus was calculated by measuring the gradient from 0 to 10% elongation of the stress-strain curve. Eight specimens per sample were tested and the data were averaged. The mechanical property of 3A-TCMBAs in hydrated status was also tested after being hydrated in water for 2 h.

### 2.8 Anti-oxidant activity evaluation

The anti-oxidant activity of 3A-TCMBA was assessed by 2,2-diphenyl-1-picrylhydrazyl (DPPH) assay, adapted from previous literature [45,46]. Typically, 2 mg of dried 3A-TCMBA was added to 3 mL DPPH (100 μM) solution in methanol. The mixture was then incubated in the dark for a desired period. Wavelength scanning of the solution was performed using a UV–vis spectrophotometer (SHIMADZU UV-2550). The DPPH scavenging percentage was calculated using the following equation [47,48].

$$DPPH\;scavenging\;\left(\%\right)\;=\frac{A_{B}-A_{S}}{A_{B}}\times 100\%$$

where A_B_ and A_S_ are the absorbances of blank (DPPH + methanol) and sample (DPPH + methanol + sample), respectively.

In addition, for the measurement of anti-oxidant enzyme activities, wound tissues treated with 3A-TCMBA were collected, and the supernatants of lysates were quantitatively assayed. The activities of superoxide dismutase (SOD), catalase (CAT), and glutathione peroxidase (GPx) were determined according to the manufacturer's instructions (Beyotime Institute of Biotechnology, China).

### 2.9 *In vitro* study

Mouse fibroblasts (L929) were incubated in Dulbecco's modified Eagle's medium (DMEM, GIBCO, Thermo Fisher Scientific), with 10% (V/V) fetal bovine serum (FBS, Ausgenex, New Zealand, Australia) and 1% (V/V) penicillin/streptomycin (Cyagen Biosciences Inc. USA). Cells were cultured in a humidified incubator (Thermo Fisher Scientific, USA) at 37 °C and 5% CO_2_. The media was changed every 2 days until the cells reached 80% confluence.

The cytocompatibility of 3A-TCMBAs was evaluated using sol content and degradation products. Cell Counting Kit-8 (CCK-8, Jiancheng Co, Nanjing, China) assay against L929 cells was conducted. For sol content, 0.5 g dried 3A-TCMBA was incubated in 5 mL PBS (pH 7.4) at 37 °C for 24 h. For degradation products, 1 g dried 3A-TCMBAs were fully degraded in 10 mL of 0.2 M NaOH solution and the pH was adjusted to 7.4. The concentration of sol contents and degradation products was diluted to 1 × , 10 × and 100 × , respectively, using PBS (pH 7.4). L929 cells were seeded at a cell density of 1 × 10^4^ cells/well with 1 mL DMEM (containing 10% FBS and 1% penicillin/streptomycin) and incubated in the wells of a 24-well cell culture plate for 24 h. Then, 0.9 mL DMEM and 100 μL sterilized sol content or degradation product with various dilutions were added, and the cells were incubated for another 24 h, followed by CCK-8 assay according to the manufacturer's procedure. Cells that were exposed to DMEM media were used as control, and the absorbance at a wavelength of 450 nm was measured. The cell viability was calculated as follows [49]:

Cell viability (%) = Ab_sample_ /Ab_control_ × 100%

The viability and morphology of cells were also observed by Live/Dead assay using acridine orange/ethidium bromide (AO/EB) double staining, using 1 × sol content solution of 3A-TC_15%_ as an example. After incubation for 1, 3, and 5 days, L929 cells were washed with PBS three times and then incubated with Live/Dead staining media for 10 min, followed by observation under an inverted fluorescent microscope (Olympus CKX41, Tokyo, Japan). The live cells were stained green, while the dead cells were stained red.

The migration of L929 cells was evaluated using a scratch assay method. Briefly, L929 cells were cultured in 6-well cell culture plates until 80% confluency before being starved in DMEM containing 10% FBS for 12 h. Then, a pipette tip was used to generate a linear wound, and the cell debris was rinsed with fresh medium. The scratched cells were cultured with fresh DMEM containing 10% FBS and different sol contents (10 × ) of 3A-TCMBAs conditioned medium for 12 and 24 h. Phase contrast images were then taken by an inverted fluorescent microscope. In addition, the cell migration ability of L929 cells was further assessed using a transwell invasion assay. Briefly, transwell chambers were put into 24 well plates, and then 500 μL fresh DMEM containing 10% FBS and different sol contents (10 × ) of 3A-TCMBAs were added. After that, 1 × 10^4^ cells/mL was added and cultured for 24 h at 37 °C. The invaded cells were fixed with 4% paraformaldehyde for 30 min and stained with crystal violet for 15 min. The images of the invaded cells were observed and photographed under an optical microscope, then the chamber was immersed in 30% glacial acetic acid for elution, and the OD value at 570 nm was measured.

### 2.10 Photothermal behavior and photothermal enhanced *in vitro* antibacterial activities of 3A-TCMBAs

The photothermal property of 3A-TCMBAs was studied with a near-infrared laser (808 nm, B0T808-5W), using 3A-TC_15%_ as an example. 3A-TC_15%_ was cut into a circular shape, and 808 nm near-infrared light was applied to the surface. The data for temperature-time curves were collected and used to evaluate the photothermal property of 3A-TCMBAs. Furthermore, the effect of power densities (0.5, 0.85, and 1.2 W/cm^2^) was also investigated. Four cycles of heating and cooling were also conducted to verify repeatable photothermal properties of 3A-TCMBAs. The heat maps and temperature profiles of the 3A-TCMBAs during light irradiation were recorded using an infrared (IR) thermal imaging camera (UTI165H).

The *in vitro* antibacterial activities of 3A-TCMBAs with or without NIR irradiation was evaluated using *E. coli* (ATCC 8739, Gram-negative) and *S. aureus* (ATCC 6538, Gram-positive) as representative bacteria. Briefly, the dried adhesive films were cut into a circular shape (∼0.1 g), then the sterilized films were added to 24-well plates. 10 μL of bacterial suspension in PBS (10^8^ colony-forming units (CFU)/mL) was added onto the surface of the adhesive film. After that, the samples were exposed to NIR laser light (808 nm, 0.5 W/cm^2^) for 10 min and compared to 3A-TCMBAs treated samples without NIR irradiation. Untreated bacterial suspension (10^8^ CFU/mL, 10 μL) on a petri dish was used as a negative control. After 10 min, 1 mL of sterilized PBS was introduced into each well to re-suspend surviving bacteria. Then, the diluted bacterial suspensions were cast uniformly on agar plates in Petri dishes and cultured for 18–24 h at 37 °C followed by CFU counting. All experiments were performed in triplicate and the results were averaged.

### 2.11 *In vivo* evaluation

#### 2.11.1 Infected full-thickness skin defect model

Wound healing evaluation of 3A-TCMBAs was performed using an infected full-thickness skin defect model on male SD rats (240–280 g), as previously reported [50]. All animal experiments were conducted in compliance with the Animal Experimental Committee of Southern Medical University (Approval No. SYXK2016-0167). Before surgery, the rats were acclimatized for 1 week. Rats were anesthetized by intraperitoneal injection of 10% chloral hydrate, followed by shaving and sterilization with iodine. Three full thickness wounds with a diameter of 1.2 cm were created on the back of one rat, and 10 μL *S. aureus* (10^8^ CFU/mL) was injected into the wound to induce wound infection [14,51]. Then, 3A-TCMBAs were applied to the wound area, and NIR (0.5 W/cm^2^) was used to irradiate one adhesive group *in situ* for 10 min. The thermographic images of the tested rats were recorded by an IR thermal camera. The rats were divided into three groups: physiological saline (control), 3A-TC_15%_+NIR (with NIR) and 3A-TC_15%_ (no NIR), and the functional wound dressing of 3A-TC_15%_ was changed every three days until wound healing, and a 10 min’ 808 nm NIR irradiation was conducted after each dressing change. The process of wound regeneration was studied by monitoring wound area, and macroscopic photographs of the treated wounds were taken on the 3rd, 7th, 14th and 21st days. The wound size was measured by Image J software, and the wound healing ratio was calculated using the following equation [52]:

$$Wound\;healing\;ratio\;\left(\%\right)\;=\frac{\left(A_{0}-A_{t}\right)}{A_{0}}\times 100\%$$

where A_0_ and A_t_ are the initial wound area and the wound area at desired time t, respectively.

#### 2.11.2 Wound closure evaluation

To evaluate the *in vivo* wound closure performance of 3A-TCMBAs, a full-thickness rat skin incision model was established using male Sprague-Dawley (SD) rats (240–280 g). All animal experiments were conducted in compliance with the Animal Experimental Committee of Southern Medical University (Approval No. SYXK2016-0167). After being paralyzed with 10 wt% chloral hydrate, the rats were fixed on a surgical corkboard, followed by shaving and sterilization with iodine. Then, four full-thickness incisions (1.5 cm in length) were created on the back of the rat, and treated with 3A-TCMBA adhesive (with NIR and without NIR) and suture, with no treatment as control. The incisions area was monitored and photographed on the 3rd, 5th, 7th, 10th and 14th day.

#### 2.11.3 Histology and immunohistochemistry evaluation

For histological examination, the wound skin tissues were isolated and immediately fixed in 4% formaldehyde in PBS at 4 °C. After being embedded in paraffin, skin tissues were sectioned into 4 μm thickness slices for hematoxylin and eosin (H & E), Masson trichrome and picrosirius red staining. All the stained histological sections were observed with an optical microscope (DMI3000B, Leica). Collagen density (%), collagen type I/III ratios, epidermis thickness and quantification of hair follicles were determined by the analysis of Masson trichrome and picrosirius red staining images using Image J software.

For immunohistochemical staining, the skin tissue sections embedded in paraffin were de-paraffinized, washed with PBS several times, and then blocked with 5% serum for 30 min. Subsequently, the slides were incubated with primary interleukin-1β (IL-1β) (Abcam), tumor necrosis factor (TNF-α) (Abcam), platelet endothelial cell adhesion molecule-1 (CD31) (Abcam), vascular endothelial growth factor (VEGF) (Abcam), platelet derived growth factor (PDGF) (Abcam), CD34 (Abcam) and matrix metalloproteinase-9 (MMP9) (Abcam) antibodies, respectively, at 4 °C overnight, followed by incubation with peroxidase-conjugated secondary antibodies. All stained histological sections were observed with an optical microscope. Inflammatory cells and vessel numbers were calculated using Image J software. At least 3 random areas were selected and the results were averaged.

### 2.12 Statistical analysis

Statistical analysis was performed by a one-tailed Student's t-test using Statistical software (SPSS). The experimental results were expressed as mean ± standard division (SD). The differences were considered statistically significant when the *p*-values were <0.05. * and ** represent *p* < 0.05 and *p* < 0.01, respectively.

## 3 Results and discussion

### 3.1 Characterizations of 3A-TCMBAs

**Fig. 1:**
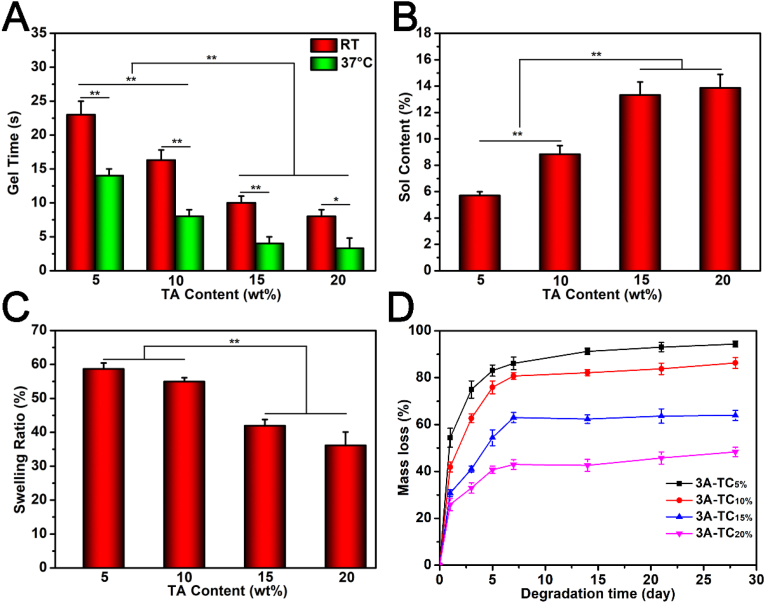
Characterizations of 3A-TCMBAs: (A) gel times, (B) sol contents, (C) swelling ratios, and (D) degradation rates. (*p < 0.05, **p < 0.01).

Gel times of 3A-TCMBAs were examined using the tilting test method. As shown in Fig. 1A, all 3A-TCMBAs displayed fast gelation (gel time <25 s) at various TA contents (5, 10, 15, and 20 wt%) at both room temperature and 37 °C, which is attributed to the fast hydrogen bonding interaction between iC-E-Ca^2+^ prepolymer and TA. Higher TA content and higher temperature all induced faster gelation. Specifically, the gel times for 3A-TC_5%_, 3A-TC_10%_, 3A-TC_15%_ and 3A-TC_20%_ were about 23.0, 16.3, 10.0 and 8.0 s, respectively, at room temperature, while the gel times for the same samples decreased to 14.0, 8.0, 4.0 and 3.3 s, respectively, when the temperature was increased to 37 °C. By adjusting TA content and polymer concentration, the gelation time can be adjusted to meet the needs of a specific tissue engineering application. Sol contents of different 3A-TCMBAs are shown in Fig. 1B. It can be seen that sol content increased proportionally with TA content. However, all sol contents were lower than 15%, indicating effective crosslinking between iC-E-Ca^2+^ and TA via hydrogen bonding. The swelling ratios of 3A-TCMBAs are shown in Fig. 1C, which decreased with increasing TA content. The lowest swelling ratio was <40 wt% (3A-TC_15%_), lower than similar hydrophobic adhesive samples chemically crosslinked by magnesium oxide, as reported in our previous work [41].

The degradation profiles of 3A-TCMBAs are shown in Fig. 1D. As can be seen, with the increase of TA content, degradation rates decreased. In detail, the 3A-TC_5%_ adhesive exhibited the fastest degradation rate in the tested formulations, with over 80% mass loss in 5 days, while the 3A-TC_20%_ adhesive exhibited the lowest degradation rate, with less than 50 wt% mass loss after 28 days. These results indicated that higher TA content could lead to higher crosslinking density and render the crosslinking system of TA and pre-polymer more stable.

### 3.2 Adhesion and self-healing properties

**Fig. 2:**
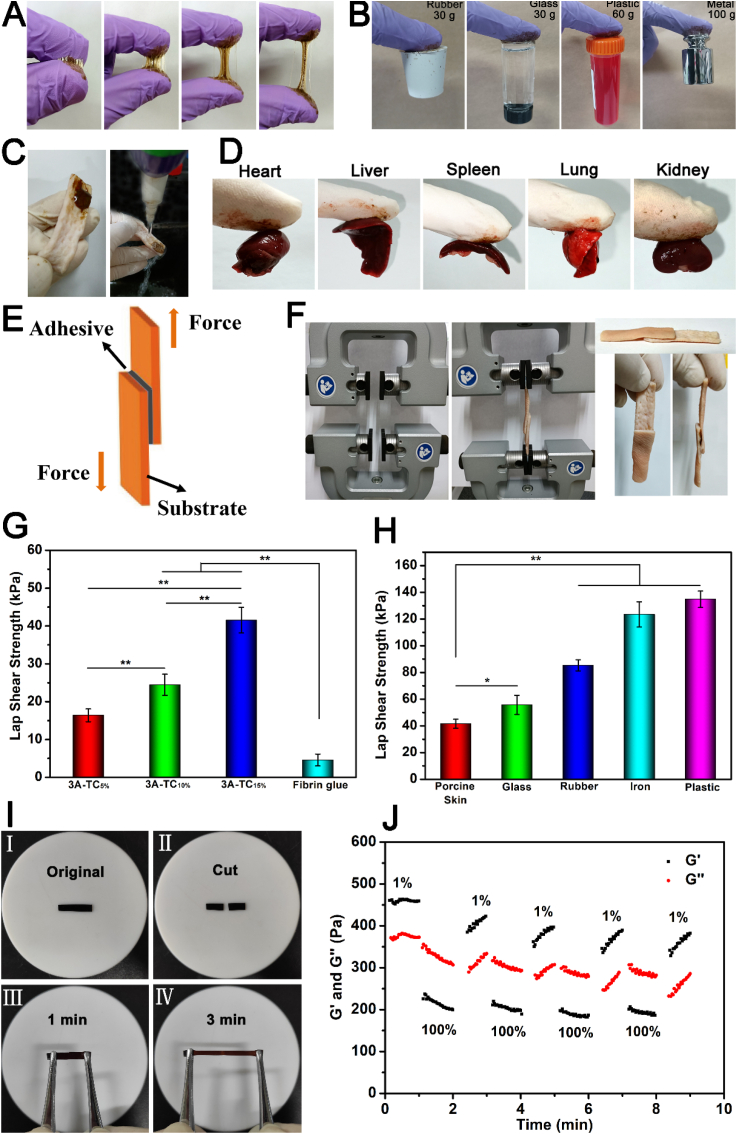
Adhesive properties of 3A-TCMBAs: (A) photographs of macroscopic adhesion, (B) photographs of the adhesion ability to glass, rubber, iron, and plastic, (C) photographs of the adhesion to porcine skin under water flushing, (D) adhesive properties to various tissues, (E, F) schematic presentation of the lap shear test, the lap shear strengths to (G) porcine skin and (H) other substrates, (I) the macroscopic photographs of self-healing capacity, (J) rheological behavior with alternate strains switched from 1% to 100% for four cycles. (*p < 0.05, **p < 0.01).

The 3A-TCMBAs showed robust adhesion to various substrates such as rubber, glass, plastic, and metal (Fig. 2A and B), indicating the possible wide applicability of the adhesive. A small piece (∼0.1 g) of 3A-TCMBA on a glove could firmly hold a weight of 30 g rubber, 30 g glass, 60 g plastic and 100 g metal (Fig. 2B). The 3A-TCMBAs also displayed strong adhesion to wet tissues including porcine skin and various visceral organs including heart, liver, spleen, lung, and kidney (Fig. 2C and D). The adhesion strengths of 3A-TCMBAs to different substrates were quantitatively investigated by lap shear strength test (Fig. 2E–H and 2F). As shown in Fig. 2G, the lap shear strengths of 3A-TCMBAs to porcine skin at wet conditions increased proportionally to TA content. The 3A-TC_5%_ (crosslinked by 5% TA) showed the lowest adhesion strength in the tested formulations (16.40 ± 1.71 kPa), while 3A-TC_15%_ adhesive displayed the highest adhesion strength (41.54 ± 3.40 kPa). The adhesion strengths of 3A-TCMBAs were higher than that of commercially available fibrin glue (4.58 ± 1.57 kPa), typically used as a gold standard tissue adhesive. As shown in Fig. 2H, the 3A-TC_15%_ also exhibited strong adhesion to glass, rubber, iron, and plastic, with the highest adhesion strength reaching ∼140 kPa (to plastic).

The fast self-healing behavior of 3A-TCMBA represented by 3A-TC_15%_ is demonstrated in Fig. 2I. Fully cut 3A-TCMBA film pieces could be fused together in 3 min by simply contacting their freshly cut interfaces without any external stimuli. The self-healing property of 3A-TC_15%_ was also quantitatively studied via rheological test. As shown in Fig. 2J, it can be seen that after relaxation for ∼1 min following 1 min of subjection to a large strain (100%), the storage modulus (Gꞌ) of 3A-TC_15%_ could be mostly restored even after 4 cycles.

The adhesive property of the 3A-TCMBAs mainly originates from the phenolic hydroxyl groups derived from dopamine (DA) and TA, which could strongly bond to various substrates through hydrogen bonding, electrostatic interaction, hydrophobic interaction, ion coordination, and even chemical reaction with amino groups of proteins on the tissue surface [53]. The existence of ionic bonds (–COO^-^---Ca^2+^----^-^OOC–) as well as hydrogen bonding (between the EPE chains or dopamine (DA) side groups of iC-E-Ca^2+^ prepolymer and the polyphenol groups on TA) confer self-healing property to 3A-TCMBAs. The strong wet tissue adhesion favors the application of 3A-TCMBAs in wound healing, and the favorable self-healing property confers 3A-TCMBAs with the ability to repair automatically upon damage in dynamic and complex wound sites.

### 3.3 Mechanical properties of 3A-TCMBAs

**Fig. 3:**
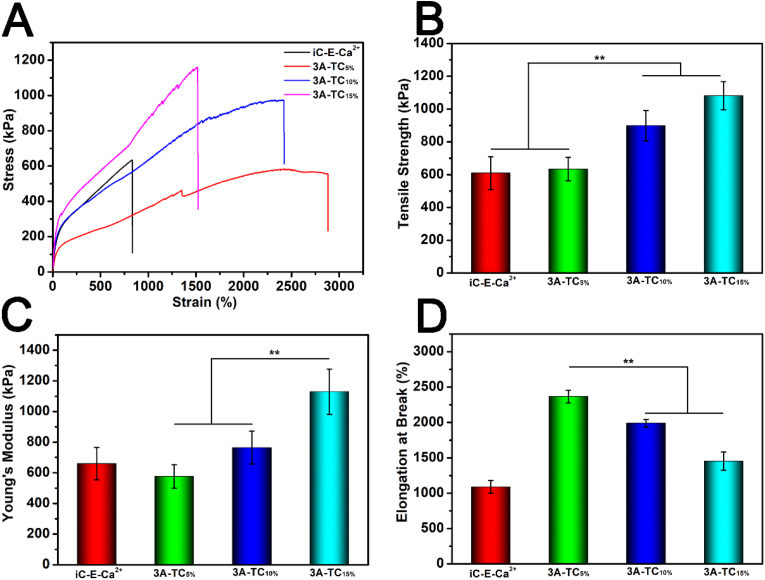
Mechanical characterizations of 3A-TCMBAs: (A) stress-strain curves, (B) tensile strengths, (C) Young's moduli, (D) elongations at break. (**p < 0.01).

The mechanical properties of dried 3A-TCMBAs were investigated by tensile test (Fig. 3). As shown in Fig. 3A, the stress-strain curves of 3A-TCMBAs all demonstrated an ultra-high elasticity. In contrast to iCMBA prepolymers untreated with calcium carbonate, the uncrosslinked iC-E-Ca^2+^ prepolymer film also exhibited an elastic property, attributed to the ion interactions between the side carboxylate anions on iC-E-Ca^2+^ prepolymer and calcium cations. The tensile strengths of 3A-TCMBAs were in the range of 550–1200 kPa, and with increased TA content, the tensile strength also increased (Fig. 3B). The Young's moduli of 3A-TCMBAs also increased gradually with TA content, while the elongation at break decreased (Fig. 3C and D). All the 3A-TCMBA films possessed elongations (1500–2500%) much higher than that of uncrosslinked iC-E-Ca^2+^ prepolymer film (∼1200%), further indicating the predominant role of hydrogen bonding in the crosslinked 3A-TCMBAs. Although the hydrogen bond interaction is a non-covalent bond, the results indicated that TA could effectively crosslink iC-E-Ca^2+^ prepolymer and make the crosslinked network more stable. In addition, the mechanical profiles of 3A-TCMBAs in hydrated status were tested and the results are shown in Table S2. As seen, 3A-TCMBAs maintain considerable mechanical strengths at hydrated and swollen status, indicating that 3A-TCMBAs have tailorable mechanical properties for various clinical applications.

### 3.4 Photothermal and antibacterial properties of 3A-TCMBAs

**Fig. 4:**
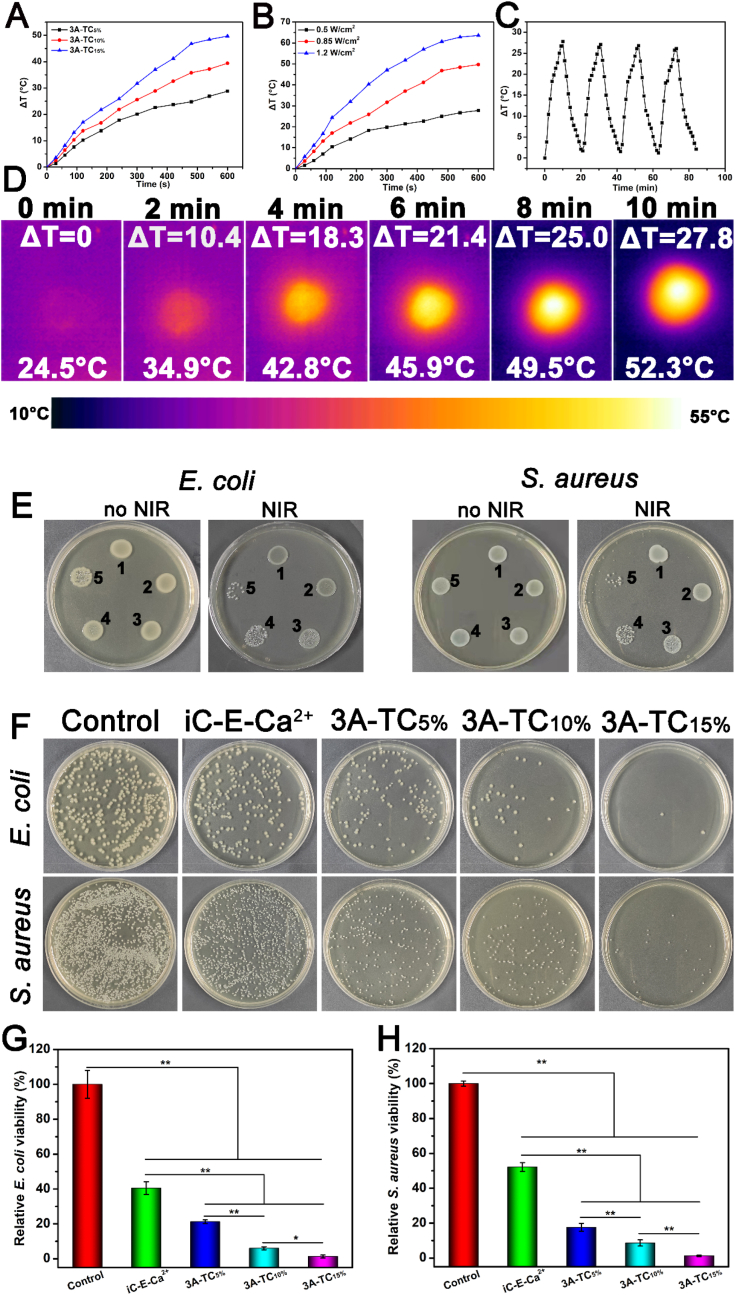
Photothermal and photothermal antibacterial properties: the curves of ΔT-NIR irradiation time for (A) 3A-TCMBAs with different TA contents at 0.85 W/cm^2^ and (B) 3A-TC_15%_ under different NIR power densities; (C) temperature change of 3A-TC_15%_ over four NIR irradiation on/off cycles (0.5 W/cm^2^); (D) the representative thermal graphs of 3A-TC_15%_ upon 808 nm NIR irradiation (0.5 W/cm^2^) for 10 min; (E, F) contacting antibacterial activity of 3A-TCMBAs, (E) bacterial colonies of *E. coli* and *S. aureus* cocultured with PBS (1), iC-E-Ca^2+^ (2), 3A-TC_5%_ (3), 3A-TC_10%_ (4) and 3A-TC_15%_ (5) and treated without or with NIR; (F) representative images of colony forming units for *E. coli* and *S. aureus* suspensions on LB agar plates following 12 h incubation at 37 °C; relative bacterial viabilities of (G) *E. coli* and (H) *S. aureus*. (*p < 0.05, **p < 0.01).

The photothermal property of 3A-TCMBAs was investigated via the temperature rise (ΔT) upon 808 nm NIR irradiation. The temperature changes of 3A-TCMBA hydrogels with different TA contents were measured upon NIR irradiation for 10 min (power density = 0.85 W/cm^2^). It could be seen that ΔT increased proportionally with TA content (Fig. 4A). From Fig. 4B, it can be seen that the temperature of 3A-TC_15%_ hydrogel increased about 25 °C (to 50 °C) within 10 min under 808 nm NIR irradiation at a power density of 0.5 W/cm^2^, whereas, almost 65 °C was reached (ΔT = 40 °C) using 1.2 W/cm^2^ NIR irradiation in the same manner. In addition, 3A-TC_15%_, as a representative example, displayed highly repeatable photothermal properties. As shown in Fig. 4C, after four cycles of NIR irradiation (0.5 W/cm^2^) and free cooling, 3A-TC_15%_ still maintained its photothermal ability. Photothermal images of 3A-TC_15%_ being irradiated for different durations shown in Fig. 4D further demonstrated the significant increase in temperature of the irradiated 3A-TC_15%_ sample.

Recently, photothermal therapy (PTT) has been widely used to cure diseases including tumors, bacterial-infected skin wounds and chronic wounds with the help of ultraviolet (UV), visible [54,55] and NIR light, with NIR light the most popular due to its larger penetration depth. Therefore, based on the photothermal property of 3A-TCMBAs, the photothermal antibacterial activity of 3A-TCMBAs was evaluated using *E. coli* and *S. aureus* as representative Gram-negative and Gram-positive bacteria (Fig. 4E–H). As shown in the representative photos (Fig. 4E and F), 3A-TCMBAs showed weak intrinsic antibacterial activity derived from both citric acid and TA without NIR irradiation [23,24,34]. However, after NIR irradiation, 3A-TCMBAs displayed obvious antibacterial activity compared to the iC-E-Ca^2+^ and PBS groups, in line with previous studies wherein bacteria are effectively killed when the temperature increases above 50 °C [56]. Moreover, with increased TA content, the antibacterial effect was significantly enhanced, with almost complete inhibition when TA content increased to 15% (Fig. 4G and H). These results indicated that the photothermal property of 3A-TCMBA is enhanced with increased TA content.

### 3.5 Anti-oxidant activity of 3A-TCMBAs *in vitro* and *in vivo*

**Fig. 5:**
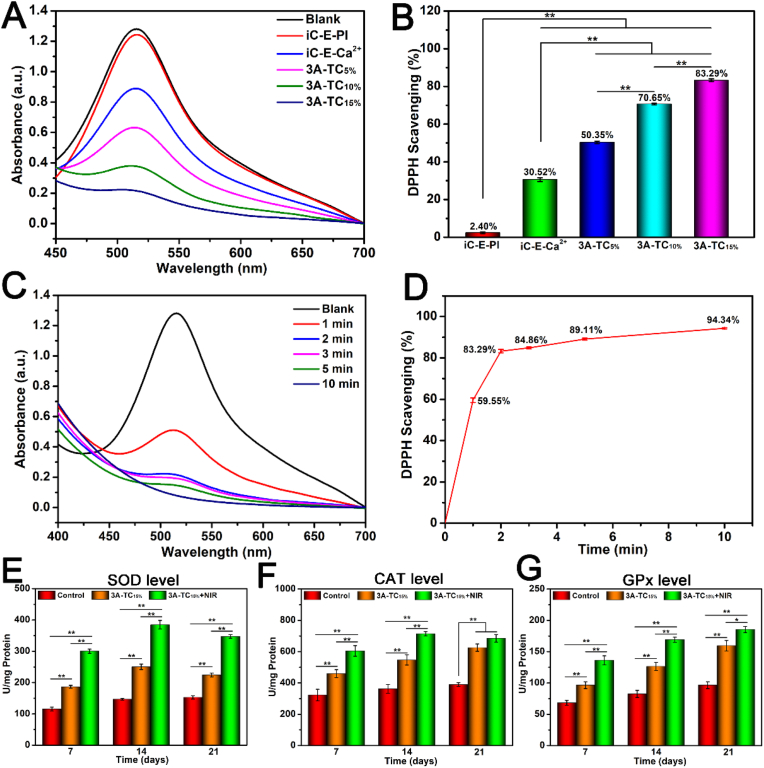
Anti-oxidant properties: (A) UV–vis spectra and (B) DPPH scavenging percentages of 3A-TCMBAs after incubating for 2 min; (C, D) dynamic DPPH scavenging of 3A-TC_15%_; expression levels of (E) superoxide dismutase (SOD), (F) catalase (CAT) and (G) glutathione peroxidase (GPx) during the process of *in vivo* wound healing after being treated with control, 3A-TC_15%_ and 3A-TC_15%_+NIR adhesive for 7, 14 and 21 days, respectively. (*p < 0.05, **p < 0.01).

Excessive ROS in the wound site can induce oxidative stress, thus delaying wound healing [57]. Both mussel-inspired dopamine (DA) and polyphenols have been reported to possess excellent anti-oxidant abilities via inhibition of the free radicals through electron transfer [58,59]. Here, the antioxidant activity of 3A-TCMBAs was evaluated by DPPH assay, one of the most accepted standards for anti-oxidant property assessment. DPPH free radicals could be neutralized by accepting an electron or hydrogen atom, leading a solution color change from purple to yellow [60,61]. As shown in Fig. 5A and B, after being treated with 3A-TCMBAs, the absorbance of DPPH at 516 nm significantly decreased, with higher TA contents leading to greater reduction. The iC-E-Ca^2+^ prepolymer also showed some anti-oxidant ability derived from DA (Fig. 5A). However, the UV–vis curve of iC-E-PI adhesive (prepared according to our previous work) was almost the same as the blank control (DPPH scavenging efficiency = 2.40%), indicating that the inclusion of strong oxidants, such as sodium periodate (PI), significantly dampened the anti-oxidant activity of iC-E-Ca^2+^ prepolymer (Fig. 5A). The DPPH scavenging efficiency of 3A-TC_15%_ reached 83.29% in 2 min, while 3A-TC_5%_ and 3A-TC_10%_ could only scavenge 50.35 and 70.65% (Fig. 5C). In addition, the scavenging efficiency increased with a longer treatment time (Fig. 5B and D). These results indicated that crosslinking with TA via hydrogen bonding rather than utilizing strong oxidant crosslinking initiators preserved the anti-oxidant activity of 3A-TCMBAs.

ROS can be eliminated by anti-oxidant enzymes *in vivo*, such as SOD, CAT and GPx [62]. Therefore the improvement of SOD, CAT and GPx expression levels is important for reducing oxidative stress and promoting rapid wound healing *in vivo* [63]. The *in vivo* anti-oxidative activity of 3A-TCMBA was also evaluated by investigating their effect on the expression of SOD, CAT and GPx in the wound site. As illustrated in Fig. 5E-G, the SOD, CAT and GPx levels for both 3A-TC15% and 3A-TC15% + NIR groups (especially the latter) were all significantly higher (*p* < 0.01) than that of the control group at the same time-points. Interestingly, on the 21st day, the levels of SOD and CAT stopped increasing compared with the 14th day, indicating a phase transfer of wound healing from inflammation to proliferation [62]. These results further confirmed the significant antioxidant ability of 3A-TCMBAs, which could effectively mitigate oxidative stress *in vivo*. Moreover, 3A-TCMBA with NIR can accelerate the wound healing process, which induces the increased expression of SOD, CAT and GPx.

### 3.6 *In vitro* cell cytocompatibility and proliferation

**Fig. 6:**
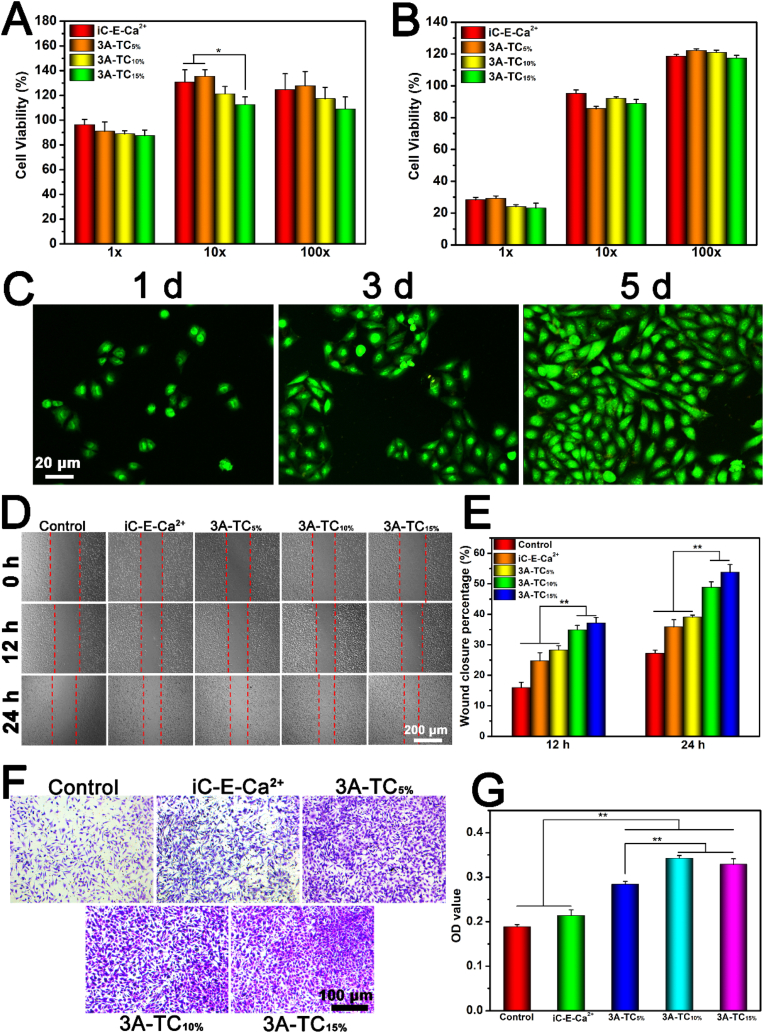
Cytotoxicity evaluation of 3A-TCMBAs: cytotoxicity against L929 cells after 24 h for (A) sol content and (B) degradation products, (C) representative Live/Dead images, (D) L929 cells migration images and (E) quantitative scratch closure rates, (F) representative images of the transwell migration assay of L929 cells and (G) quantitative migration cell OD values. (*p < 0.05, **p < 0.01).

The cytocompatibility of 3A-TCMBAs was assessed by investigating the cytotoxicity of the sol content and degradation products of 3A-TCMBAs via CCK-8 and Live/Dead assay against L929 cells. As shown in Fig. 6A, the sol content of 3A-TCMBAs at the 1 × dilution demonstrated almost no cytotoxicity, with cell viabilities all higher than 80%. Moreover, the cell viabilities increased for 10 × and 100 × diluted sol contents, with cell viabilities even higher than 100%. The 1 × degradation products of 3A-TCMBAs displayed low cell viabilities (<30%), while the cell viabilities of the 10 × and 100 × diluted degradation products became much higher (>80%), as shown in Fig. 6B. The growth of L929 cells was also investigated by the Live/Dead assay, using the 10 × sol content of 3A-TC_15%_. From Fig. 6C, cells continually proliferated over five days of culture, with almost no dead cells found. These results indicated that 3A-TCMBAs exhibited satisfactory cytocompatibility. It has been reported that, as a natural polyphenol, TA can promote the proliferation of mammalian cells [38,64,65]. The use of abundant TA rather than toxic oxidants in 3A-TCMBAs was thus expected to result in favorable cytocompatibility, duly demonstrated above.

The migration of fibroblasts is beneficial for wound healing; therefore, the effect of 3A-TCMBAs on the migration ability of L929 cells was studied via both 2D scratch assay and 3D *trans*-well assay [66]. As shown in Fig. 6D and E, the L929 cells incubated in the 10 × sol content of 3A-TCMBAs moved into the scratched area faster than the control and iC-E-Ca^2+^ (10 × sol content) groups, with cell migration rates for 3A-TC_10%_ and 3A-TC_15%_ significantly higher compared to the control and iC-E-Ca^2+^ groups (*p* < 0.01) at both 12 h and 24 h after treatment. With increasing TA content, the scratches became much narrower after 24 h. The beneficial effect of 3A-TCMBAs on the migration of L929 cells was further confirmed by the qualitative (Fig. 6F) and quantitative (Fig. 6G) data of the 3D *trans*-well study. Studies have shown that the catechol group of dopamine and the galloyl groups on TA can form a strong interaction with the imidazoles or thiols on the cytomembranes of fibroblasts [62,67]. Thus, wound healing progress might be accelerated through sustained TA and dopamine release from the materials by promoting cell proliferation and migration.

### 3.7 *In vivo* evaluation

#### 3.7.1 *In vivo* infected wound healing performance

**Fig. 7:**
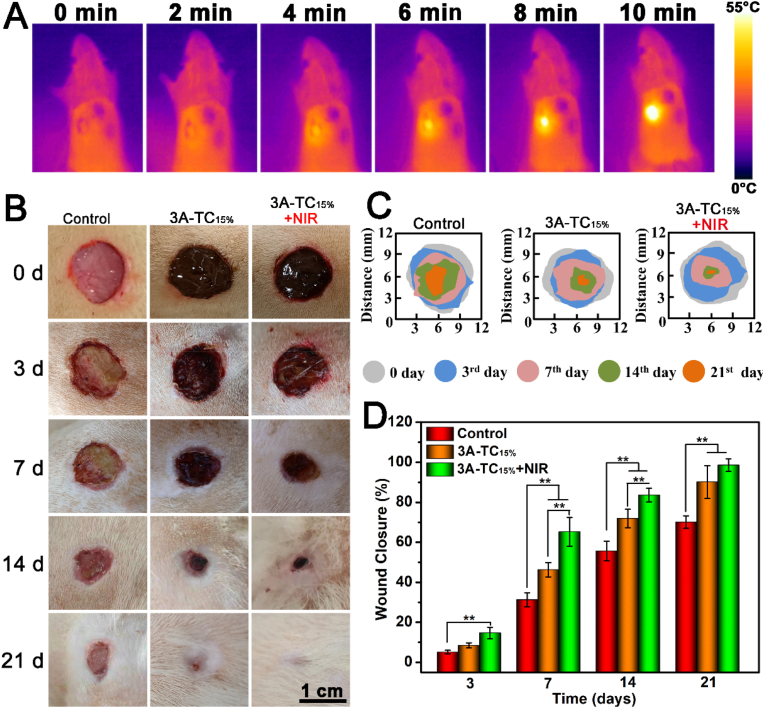
Wound closure results of the infected wounds: (A) Photothermal temperature change of SD rats for 3A-TC_15%_, 3A-TC_15%_+NIR and the control groups; (B) representative photographs of the wounds treated with physiological saline (control) and 3A-TC_15%_ (with and without NIR) on the 3rd, 7th, 14th and 21st day; (C) schematic diagram of wound closure; (D) quantitative statistical analysis of wounds closure. (**p < 0.01).

*In vitro* studies well demonstrated the tissue adhesion ability, ultra-high elasticity, self-healing property, favorable anti-oxidant activity, NIR photothermal antimicrobial activity and excellent cytocompatibility of 3A-TCMBAs, which all strongly suggested the application potential of 3A-TCMBAs in infected wound healing. Thus, the suitability of 3A-TCMBAs for skin wound healing applications was further evaluated in an infected full-thickness skin defect wound model. The photothermal temperature change images of SD rats for 3A-TC_15%_, 3A-TC_15%_+NIR and the control groups are shown in Fig. 7A. The infected wound-site temperature in the 3A-TC_15%_+NIR group rapidly increased within 5 min upon NIR irradiation, consistent with the *in vitro* results. Representative photographs of the infected wounds treated by different samples are shown in Fig. 7B. Some yellow pus around the wound could be found on the 3rd day, indicating the successful creation of an infected wound model. Furthermore, the wound areas in the 3A-TC_15%_ and 3A-TC_15%_+NIR groups closed much faster than that of the control group. On day 21, the wounds in the 3A-TC_15%_+NIR group were almost closed, while wounds in the control group remained unhealed and a scar was left, which could also be clearly seen in the overlapped wound areas of different time-points shown in Fig. 7C. The wound closure ratios of different groups were also quantitatively calculated by Image J. As seen in Fig. 7D, the average wound closure ratios of both the 3A-TC_15%_ and 3A-TC_15%_+NIR groups were significantly higher than that of the control group, especially on the 7th, 14th and 21st day (*p* < 0.01), and the wound closure ratios of 3A-TC_15%_+NIR group at day 7 and 14 were also significantly higher than that of 3A-TC_15%_ group (*p* < 0.01). These results confirmed that the application of 3A-TCMBAs and the assistance of NIR irradiation could greatly promote infected wound healing.

#### 3.7.2 Histological and immunohistochemical analysis

**Fig. 8:**
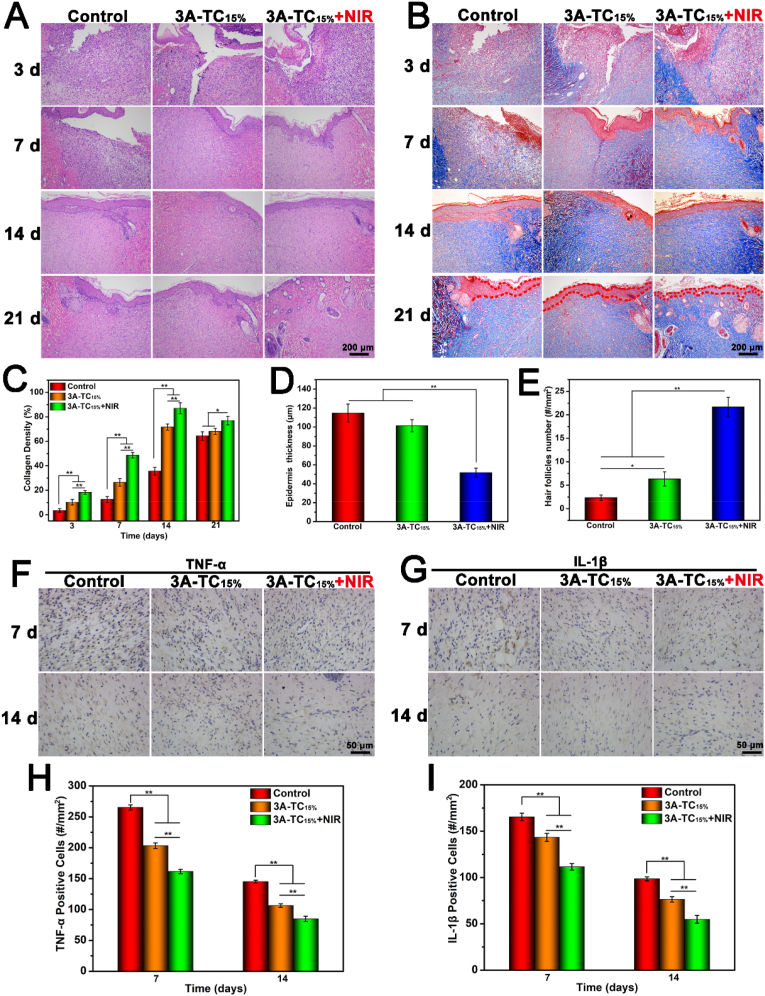
Histological and inflammation-related immunohistochemical analysis: (A) H & E and (B) Masson's trichrome staining images of the infected skin wound after being treated with 3A-TCMBAs (with and without NIR), and physiological saline (control), (C) collagen densities, (D) epidermis thicknesses (day 21) and (E) quantitative hair follicle numbers (day 21); Immunohistochemistry staining images of (F) TNF-α, and (G) quantitative IL-1β, cell densities of (H) TNF-α positive cells and (I) IL-1β positive cells. (*p < 0.05, **p < 0.01).

The wound healing efficacy of 3A-TCMBAs was further evaluated by Hematoxylin-eosin (H & E) and Masson's trichrome staining [68]. As can be seen in Fig. 8, prominent inflammation was observed in all groups on the 3rd day. On the 7th day, obvious inflammation still presented in the control group but was significantly decreased in both 3A-TC_15%_ and 3A-TC_15%_+NIR groups, particularly in the latter, which was mainly attributed to the excellent antibacterial and anti-oxidant activity derived from 3A-TCMBAs. Meanwhile, epidermis gradually formed on the 14th day, and it could be seen that a complete epidermis was regenerated in both 3A-TC_15%_ and 3A-TC_15%_+NIR groups but not in the control group (Fig. 8A). On the 21st day, the epidermis in all groups was completely regenerated and began to form some skin appendages, such as hair follicles and blood vessels, in the dermis, especially in the two adhesive groups (Fig. 8A).

The formation of collagen at the wound site plays a vital role in remodeling damaged skin tissue and was assessed by Masson's trichrome staining [69]. As shown in Fig. 8B, collagen deposition could be seen in all groups at all tested time points. Less collagen deposition was found in the control group, while the adhesive groups (especially 3A-TC_15%_+NIR) showed more collagen deposition with a better organized fibrous structure (Fig. 8B). The quantitative analysis results shown in Fig. 8C further revealed that the collagen densities in the 3A-TC_15%_+NIR group were significantly higher than that of the 3A-TC_15%_ group (*p* < 0.05) on the 7th and 14th day, and they were all significantly higher than that of the control groups at the same time-points. In addition, to evaluate the collagen subtype progression, picrosirius red staining was performed (staining collagen I in red and collagen III in green) as the collagen I/III ratio is often used to evaluate scarless wound healing [70,71]. As displayed in Figs. S1A and S1B, the collagen I/III ratios of the adhesive groups (especially 3A-TC_15%_+NIR) were significantly lower than that of the control group (*p* < 0.01). The collagen I/III ratio was 38.56 ± 3.54 for the control group, while it decreased to 8.96 ± 1.45 and 4.32 ± 0.56 for the 3A-TC_15%_ and 3A-TC_15%_+NIR groups, respectively. This result indicated that 3A-TC_15%_ could improve scarless wound healing. Quantitative epidermis thickness results are illustrated in Fig. 8D, revealing that epidermis thickness decreased in the adhesive groups (especially 3A-TC_15%_+NIR) compared to the control group, further implying decreased scar tissue formation in the adhesive groups (especially 3A-TC_15%_+NIR) [70]. Finally, the quantification of hair follicles was studied, as shown in Fig. 8E. Regenerated skin in the adhesive groups (especially 3A-TC_15%_+NIR) was filled with many regenerated hair follicles and sebaceous glands, further confirming that 3A-TC_15%_ leads to scarless and effective regeneration. These results suggested that 3A-TCMBAs (especially with NIR assistance) could greatly enhance collagen deposition and ordered arrangement, thus inhibiting scar formation during the wound healing process.

Acute inflammatory responses may lead to excessive oxidant pressure, causing tissue damage and delayed wound healing [65], while the intrinsic anti-oxidant activity of TA is believed to impart a considerable anti-inflammatory ability to the 3A-TCMBAs. Therefore, two representative pro-inflammatory factors, interleukin-1β (IL-1β) and tumor necrosis factor-α (TNF-α), were detected to estimate the efficacy of 3A-TCMBAs in preventing infection and extenuating inflammation during wound healing. The expression of the two cytokines was reduced in both 3A-TC_15%_ and 3A-TC_15%_+NIR groups (Fig. 8F, G, 8H and 8I). Also, it could be seen that inflammatory cell numbers gradually decreased during the tissue healing process. On the 14th day, there was no significant inflammatory response in all groups, as indicated by the mild expression levels of IL-1β and TNF-α (Fig. 8F, G, 8H and 8I). These results suggested that the 3A-TCMBAs (especially with the assistance of NIR) could effectively prevent wound infection and reduce the inflammatory response due to the excellent anti-oxidant and photothermal antibacterial properties of 3A-TCMBAs.

**Fig. 9:**
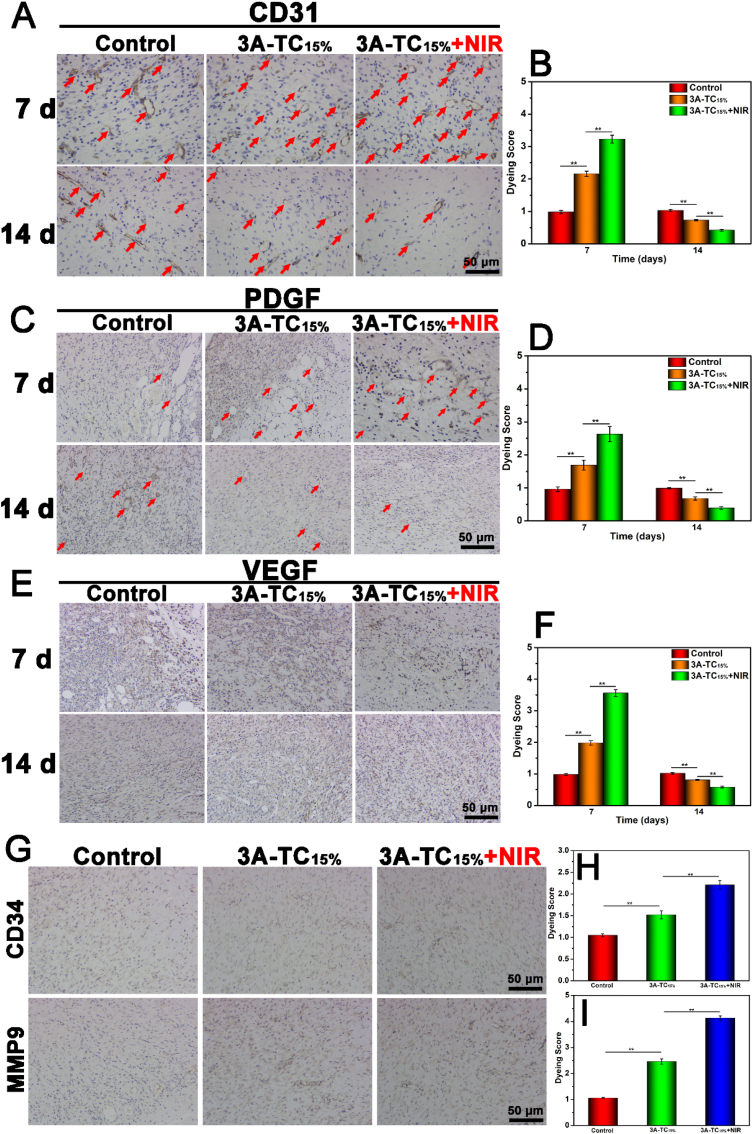
Angiogenesis, hair follicle and remodeling related immunohistochemical analysis: Immunohistochemical staining images and the corresponding dyeing scores of (A, B) CD31 (Red arrows represent new blood vessels), (C, D) PDGF, (E, F) VEGF at day 7 and 14; immunohistochemistry staining images and the corresponding dyeing scores after being treated of (G, H, I) of CD34 and MMP9 on day 21. (**p < 0.01).

In addition, the effect of 3A-TCMBAs on the neovascularization of the wound sites was also evaluated by immunohistochemical staining of CD31, PDGF, and VEGF on the 7th and 14th day. As shown in Fig. 9A-F, the expression of CD31, VEGF and PDGF in the adhesive groups, especially in the 3A-TC_15%_+NIR group, were all significantly higher than that of the control group (*p* < 0.01) on the 7th day. The enhanced expression of endovascular factors is favorable for angiogenesis and could promote wound healing in the early stage. On the contrary, after being treated with 3A-TC_15%_ and 3A-TC_15%_+NIR for 14 days, the expression levels of CD31, PDGF and VEGF all became lower than that of the control group (*p* < 0.01), which is beneficial to promote scarless skin formation at the later stage (remodeling stage) of wound healing. Studies have shown that timely degeneration of the temporary built immature blood vessels is necessary for avoiding scar formation [72]. Therefore, the opportune and phased angiogenesis ability of 3A-TCMBAs is believed to accelerate wound closure and promote scarless wound healing by inhibiting excessive neovascularization and subsequent over deposition of collagen in the remodeling phase. To further evaluate scarless wound healing efficacy, the expression of CD34 (a hair follicle stem cell marker) and MMP9 (matrix metalloproteinase) in the wound sites on the 21st day was studied [73]. As shown in Fig. 9G and H, the expression levels of CD34 and MMP9 in the 3A-TC_15%_+NIR group were significantly higher than that of the 3A-TC_15%_ group, and expression levels in the two adhesive groups were significantly higher than that of the control group. High expression levels of CD34 implied more skin appendage (such as hair follicle) regeneration, implying that the adhesive groups can stimulate hair regeneration, especially with the assistance of NIR. This is reinforced by the appearance of hair follicles in the H & E and Masson's trichrome staining images shown in Fig. 8A and B. Higher expression levels of MMP9 in the adhesive groups indicated that the 3A-TCMBAs could induce more active extracellular matrix degradation, especially the degradation of collagen I, in the remodeling phase of wound healing, thus inhibiting scar formation and promoting scarless wound healing [73].

#### 3.7.3 Wound closure study on a skin incision model

**Fig. 10:**
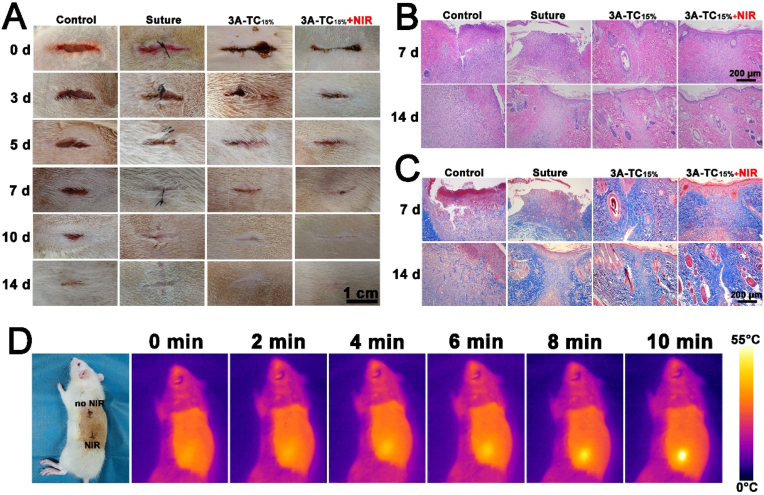
Wound closure assessment of skin incision: (A) representative images of untreated skin incisions and the skin incisions treated by surgical sutures, 3A-TC_15%_ (with and without NIR); (B) H & E staining images and (C) Masson's trichrome staining of the skin incisions on the 7th and 14th day; (D) photothermal temperature change images of SD rats for 3A-TC_15%_ and 3A-TC_15%_+NIR groups.

The tissue adhesion and wound closure performance of 3A-TCMBAs was further assessed in a full-thickness skin incision model *in vivo*. Skin incisions (1.5 cm) were made on the back of SD rats, and the incisions were closed with surgical suture or 3A-TC_15%_ with or without NIR irradiation, with the untreated wound as control. As illustrated in Fig. 10A, the incisions treated by surgical sutures and 3A-TCMBAs were closed on day 7, while skin incisions still existed in the untreated group. After 14 days, all the incisions were almost healed. The incisions of the control and suture groups showed obvious scars after healing, while the groups treated with 3A-TCMBA showed significantly less scar formation.

Histological analysis was performed on days 7 and 14 to further evaluate the wound healing efficacy, as shown in Fig. 10. H&E staining demonstrated that both control and surgical suture groups displayed severe structural disorder and acute fibrosis on the 7th day (Fig. 10B) [22,74]. By contrast, 3A-TC_15%_ and 3A-TC_15%_+NIR groups illustrated milder inflammatory responses and decreased fibrosis (Fig. 10B). On the 14th day, 3A-TC_15%_ groups almost recovered, with highly integrated epithelium and more hair follicles, while histologic fibrosis was alleviated, especially for the 3A-TC_15%_+NIR group (Fig. 10B). Additionally, Masson's trichrome staining for collagen was performed to evaluate tissue reconstruction [75]. As shown in Fig. 10C, on the 7th day, poor tissue reconstruction was observed in the control and surgical suture groups, while 3A-TC_15%_ and 3A-TC_15%_+NIR groups showed more collagen deposition. On the 14th day, all groups displayed dense collagen fiber formation, while the collagen fibers in 3A-TC_15%_ and 3A-TC_15%_+NIR groups were significantly denser and better arranged, especially for the 3A-TC_15%_+NIR group. These encouraging results may originate from the anti-oxidant, anti-inflammatory and antibacterial activities of DA and TA in 3A-TCMBAs, as well as their favorable photothermal activity. The wound closure study further demonstrated the potential of 3A-TCMBAs in scarless wound healing [76,77].

## 4 Conclusion

In this work, the abundant hydrogen bonding capability of the naturally derived anti-oxidant tannic acid was utilized as a novel crosslinking agent for injectable citrate-based mussel-inspired bioadhesive (iCMBA) prepolymer, generating a family of anti-oxidant, anti-inflammatory and antibacterial tannin-crosslinked citrate-based mussel-inspired bioadhesives (3A-TCMBAs). Elimination of the strong oxidants traditionally used in mussel-inspired bioadhesive crosslinking preserved the dopamine-derived anti-oxidant activity of iCMBA, which was further enhanced via the inclusion of TA, endowing the adhesive with significant anti-inflammatory capability. 3A-TCMBAs achieved rapid gelation, low swelling ratios, super-high elasticity, strong wet tissue adhesiveness, self-healing ability, excellent anti-oxidant properties and considerable biocompatibility. Further, the innate antimicrobial property of TA was reinforced by NIR-assisted photothermal ablation, synergistically enhancing the antibacterial effect. Convergence of the above properties led to significantly accelerated *in vivo* wound healing in both infected full-thickness and skin incision wound models. Most significantly, 3A-TCMBAs (with the assistance of NIR) could promote scarless wound healing by enabling phased angiogenesis (enhanced in the early wound healing phase but diminished in the later phases) and triggering late stage degradation & remodeling of the extracellular matrix. The design principles of 3A-TCMBAs can be universally expanded to other polymeric systems, inspiring a new methodolgy for the development of next-generation adhesives with enhanced clinical outcomes.

## Ethics approval and consent to participate

All animal experiments were conducted in compliance with the Animal Experimental Committee of Southern Medical University (Approval No. SYXK2016-0167).

## CRediT authorship contribution statement

**Keke Wu:** Conception, Methodology, Experiment conduction, Data presentation, Analysis, Writing. **Meimei Fu:** Polymer synthesis, Antioxidant, photothermal antimicrobial experiments conduction, Software. **Yitao Zhao:** Animal study and histological examination. **Ethan Gerhard:** Revision. **Yue Li:** Help on antimicrobial experiments. **Jian Yang:** Revision, Edit. **Jinshan Guo:** Supervision, Conception, Methodology, Revision.

## Declaration of interest

The authors declare that they have no known competing financial interests or personal relationships that could have appeared to influence the work reported in this paper.

## References

[1] Zhou L., Zheng H., Liu Z., Wang S., Liu Z., Chen F., Zhang H., Kong J., Zhou F., Zhang Q. (2021). Conductive antibacterial hemostatic multifunctional scaffolds based on ti_3_c_2_t_x_ mxene nanosheets for promoting multidrug-resistant bacteria-infected wound healing. ACS Nano.

[2] Fan Z., Deng J., Li P.Y. (2019). A new class of biological materials: cell membrane-derived hydrogel scaffolds. Biomaterials.

[3] Luo M., Wang M., Niu W., Chen M., Cheng W., Zhang L., Xie C., Wang Y., Guo Y., Leng T. (2021). Injectable self-healing anti-inflammatory europium oxide-based dressing with high angiogenesis for improving wound healing and skin regeneration. Chem. Eng. J..

[4] Ji D., Zhang Y., Zang Y., Li J., Chen G., He X., Tian H. (2016). Targeted intracellular production of reactive oxygen species by a 2D molybdenum disulfide glycosheet. Adv. Mater..

[5] Zhang S., Li Y., Qiu X., Jiao A., Luo W., Lin X., Zhang X., Zhang Z., Hong J., Cai P. (2021). Incorporating redox-sensitive nanogels into bioabsorbable nanofibrous membrane to acquire ROS-balance capacity for skin regeneration. Bioact. Mater..

[6] Le Thi P., Lee Y., Tran D.L., Thi T.T.H., Kang J.I., Park K.M., Park K.D. (2020). In situ forming and reactive oxygen species-scavenging gelatin hydrogels for enhancing wound healing efficacy. Acta Biomater..

[7] Wu H., Li F., Shao W., Gao J., Ling D. (2019). Promoting angiogenesis in oxidative diabetic wound microenvironment using a nanozyme-reinforced self-protecting hydrogel. ACS Cent. Sci..

[8] Cano Sanchez M., Lancel S., Boulanger E., Neviere R. (2018). Targeting oxidative stress and mitochondrial dysfunction in the treatment of impaired wound healing: a systematic review. Antioxidants.

[9] Ju H.W., Lee O.J., Lee J.M., Moon B.M., Park H.J., Park Y.R., Lee M.C., Kim S.H., Chao J.R., Ki C.S. (2016). Wound healing effect of electrospun silk fibroin nanomatrix in burn-model. Int. J. Biol. Macromol..

[10] Li Z., Huang X., Lin L., Jiao Y., Zhou C., Liu Z. (2021). Polyphenol and Cu2+ surface-modified chitin sponge synergizes with antibacterial, antioxidant and pro-vascularization activities for effective scarless regeneration of burned skin. Chem. Eng. J..

[11] Kong X., Fu J., Shao K., Wang L., Lan X., Shi J. (2019). Biomimetic hydrogel for rapid and scar-free healing of skin wounds inspired by the healing process of oral mucosa. Acta Biomater..

[12] Wu H., Li F., Wang S., Lu J., Li J., Du Y., Sun X., Chen X., Gao J., Ling D. (2018). Ceria nanocrystals decorated mesoporous silica nanoparticle based ROS-scavenging tissue adhesive for highly efficient regenerative wound healing. Biomaterials.

[13] Zhang C., Niu J., Chong Y., Huang Y., Chu Y., Xie S., Jiang Z., Peng L. (2016). Porous microspheres as promising vehicles for the topical delivery of poorly soluble asiaticoside accelerate wound healing and inhibit scar formation in vitro & in vivo. Eur. J. Pharm. Biopharm..

[14] Chen C., Yang X., Li S., Zhang C., Ma Y., Ma Y., Gao P., Gao S., Huang X. (2021). Tannic acid-thioctic acid hydrogel: a novel injectable supramolecular adhesive gel for wound healing. Green Chem..

[15] Gao X., Xu Z., Liu G., Wu J. (2021). Polyphenols as a versatile component in tissue engineering. Acta Biomater..

[16] Peng Y., He D., Ge X., Lu Y., Chai Y., Zhang Y., Mao Z., Luo G., Deng J., Zhang Y. (2021). Construction of heparin-based hydrogel incorporated with Cu5.4O ultrasmall nanozymes for wound healing and inflammation inhibition. Bioact. Mater..

[17] Li Y., Yu P., Wen J., Sun H., Wang D., Liu J., Li J., Chu H. (2021). Nanozyme-based stretchable hydrogel of low hysteresis with antibacterial and antioxidant dual functions for closely fitting and wound healing in movable parts. Adv. Funct. Mater..

[18] Li S., Chen A., Chen Y., Yang Y., Zhang Q., Luo S., Ye M., Zhou Y., An Y., Huang W. (2020). Lotus leaf inspired antiadhesive and antibacterial gauze for enhanced infected dermal wound regeneration. Chem. Eng. J..

[19] Han L., Lu X., Liu K., Wang K., Fang L., Weng L., Zhang H., Tang Y., Ren F., Zhao C. (2017). Mussel-inspired adhesive and tough hydrogel based on nanoclay confined dopamine polymerization. ACS Nano.

[20] Wang Y., Zhou P., Xiao D., Zhu Y., Zhong Y., Zhang J., Sui X., Feng X., Xu H., Mao Z. (2019). Chitosan-bound carboxymethylated cotton fabric and its application as wound dressing. Carbohydr. Polym..

[21] Huang S., Liu H., Liao K., Hu Q., Guo R., Deng K. (2020). Functionalized GO nanovehicles with nitric oxide release and photothermal activity-based hydrogels for bacteria-infected wound healing. ACS Appl. Mater. Interfaces.

[22] Sun F., Bu Y., Chen Y., Yang F., Yu J., Wu D. (2020). An injectable and instant self-healing medical adhesive for wound sealing. ACS Appl. Mater. Interfaces.

[23] Guo J., Kim G.B., Shan D., Kim J.P., Hu J., Wang W., Hamad F.G., Qian G., Rizk E.B., Yang J. (2017). Click chemistry improved wet adhesion strength of mussel-inspired citrate-based antimicrobial bioadhesives. Biomaterials.

[24] Guo J., Wang W., Hu J., Xie D., Gerhard E., Nisic M., Shan D., Qian G., Zheng S., Yang J. (2016). Synthesis and characterization of anti-bacterial and anti-fungal citrate-based mussel-inspired bioadhesives. Biomaterials.

[25] Kastrup C.J., Nahrendorf M., Figueiredo J.L. (2012). Painting blood vessels and atherosclerotic plaques with an adhesive drug depot. P. Natl. Acad. Sci. USA.

[26] Yuan X., Zhao Y., Li J., Chen X., Lu Z., Li L., Guo J. (2021). Citrate-based mussel-inspired magnesium whitlockite composite adhesives augmented bone-to-tendon healing. J. Mater. Chem. B.

[27] Hussain M., Suo H., Xie Y., Wang K., Wang H., Hou Z., Gao Y., Zhang L., Tao J., Jiang H. (2021). Dopamine-substituted multidomain peptide hydrogel with inherent antimicrobial activity and antioxidant capability for infected wound healing. ACS Appl. Mater. Interfaces.

[28] Zhang H., Sun X., Wang J., Zhang Y., Dong M., Bu T., Li L., Liu Y., Wang L. (2021). Multifunctional injectable hydrogel dressings for effectively accelerating wound healing: enhancing biomineralization strategy. Adv. Funct. Mater..

[29] Xie D., Guo J., Mehdizadeh M.R., Tran R.T., Chen R., Sun D., Qian G., Jin D., Bai X., Yang J. (2015). Development of injectable citrate-based bioadhesive bone implants. J. Mater. Chem. B.

[30] Ejima H., Richardson J.J., Liang K., Best J.P., van Koeverden M.P., Such G.K., Cui J., Caruso F. (2013). One-step assembly of coordination complexes for versatile film and particle engineering. Science.

[31] Kaczmarek B. (2020). Tannic acid with antiviral and antibacterial activity as a promising component of biomaterials-A minireview. Materials.

[32] Jia Z., Lv X., Hou Y., Wang K., Ren F., Xu D., Wang Q., Fan K., Xie C., Lu X. (2021). Mussel-inspired nanozyme catalyzed conductive and self-setting hydrogel for adhesive and antibacterial bioelectronics. Bioact. Mater..

[33] Liu J., Deng Y., Fu D., Yuan Y., Li Q., Shi L., Wang G., Wang Z., Wang L. (2021). Sericin microparticles enveloped with metal-organic networks as a pulmonary targeting delivery system for intra-tracheally treating metastatic lung cancer. Bioact. Mater..

[34] Guo J., Sun W., Kim J.P., Lu X., Li Q., Lin M., Mrowczynski O., Rizk E.B., Cheng J., Qian G. (2018). Development of tannin-inspired antimicrobial bioadhesives. Acta Biomater..

[35] Yang L., Han L., Jia L. (2016). A novel platelet-repellent polyphenolic surface and its micropattern for platelet adhesion detection. ACS Appl. Mater. Interfaces.

[36] Kim K., Shin M., Koh M.Y., Ryu J.H., Lee M.S., Hong S., Lee H. (2015). TAPE: a medical adhesive inspired by a ubiquitous compound in plants. Adv. Funct. Mater..

[37] Qiao Z., Lv X., He S., Bai S., Liu X., Hou L., He J., Tong D., Ruan R., Zhang J. (2021). A mussel-inspired supramolecular hydrogel with robust tissue anchor for rapid hemostasis of arterial and visceral bleedings. Bioact. Mater..

[38] Yu Y., Li P., Zhu C., Ning N., Zhang S., Vancso G.J. (2019). Multifunctional and recyclable photothermally responsive cryogels as efficient platforms for wound healing. Adv. Funct. Mater..

[39] Zhang W., Besford Q.A., Christofferson A.J., Charchar P., Richardson J.J., Elbourne A., Kempe K., Hagemeyer C.E., Field M.R., McConville C.F. (2020). Cobalt-directed assembly of antibodies onto metal-phenolic networks for enhanced particle targeting. Nano Lett..

[40] Kang J., Bai G., Ma S., Liu X., Ma Z., Guo X., Wang X., Dai B., Zhou F., Jia X. (2019). On-site surface coordination complexation via mechanochemistry for versatile metal-phenolic networks films. Adv. Mater. Interfac..

[41] Lu X., Shi S., Li H., Gerhard E., Lu Z., Tan X., Li W., Rahn K.M., Xie D., Xu G. (2020). Magnesium oxide-crosslinked low-swelling citrate-based mussel-inspired tissue adhesives. Biomaterials.

[42] Han N., Xu Z., Cui C., Li Y., Liu W. (2020). A Fe3+-crosslinked pyrogallol-tethered gelatin adhesive hydrogel with antibacterial activity for wound healing. Biomater. Sci..

[43] Li Y., Xu T., Tu Z., Dai W., Xue Y., Tang C., Gao W., Mao C., Lei B., Lin C. (2020). Bioactive antibacterial silica-based nanocomposites hydrogel scaffolds with high angiogenesis for promoting diabetic wound healing and skin repair. Theranostics.

[44] Gonsalves A., Tambe P., Le D., Thakore D., Wadajkar A., Yang J., Nguyen K.T., Menon J. (2021). Synthesis and characterization of a novel pH-responsive drug-releasing nanocomposite hydrogel for skin cancer therapy and wound healing. J. Mater. Chem. B.

[45] Qu J., Zhao X., Liang Y., Zhang T., Ma P.X., Guo B. (2018). Antibacterial adhesive injectable hydrogels with rapid self-healing, extensibility and compressibility as wound dressing for joints skin wound healing. Biomaterials.

[46] Sivakami M., Renuka R., Thilagavathi T. (2020). Green synthesis of magnetic nanoparticles via cinnamomum verum bark extract for biological application. J. Environ. Chem. Eng..

[47] Qu J., Zhao X., Liang Y., Xu Y., Ma P.X., Guo B. (2019). Degradable conductive injectable hydrogels as novel antibacterial, anti-oxidant wound dressings for wound healing. Chem. Eng. J..

[48] Zhao X., Wu H., Guo B., Dong R., Qiu Y., Ma P.X. (2017). Antibacterial anti-oxidant electroactive injectable hydrogel as self-healing wound dressing with hemostasis and adhesiveness for cutaneous wound healing. Biomaterials.

[49] Zeng Q., Qian Y., Huang Y., Ding F., Qi X., Shen J. (2021). Polydopamine nanoparticle-dotted food gum hydrogel with excellent antibacterial activity and rapid shape adaptability for accelerated bacteria-infected wound healing. Bioact. Mater..

[50] Wu K., Wu X., Chen M., Wu H., Jiao Y., Zhou C. (2020). H2O2-responsive smart dressing for visible H2O2 monitoring and accelerating wound healing. Chem. Eng. J..

[51] Wei S., Xu P., Yao Z., Cui X., Lei X., Li L., Dong Y., Zhu W., Guo R., Cheng B. (2021). A composite hydrogel with co-delivery of antimicrobial peptides and platelet-rich plasma to enhance healing of infected wounds in diabetes. Acta Biomater..

[52] Liu W., Wang M., Cheng W., Niu W., Chen M., Luo M., Xie C., Leng T., Zhang L., Lei B. (2021). Bioactive antiinflammatory antibacterial hemostatic citrate-based dressing with macrophage polarization regulation for accelerating wound healing and hair follicle neogenesis. Bioact. Mater..

[53] Han N., Xu Z., Cui C., Li Y., Liu W. (2020). A Fe3+-crosslinked pyrogallol-tethered gelatin adhesive hydrogel with antibacterial activity for wound healing. Biomater. Sci..

[54] Mao C., Xiang Y., Liu X., Cui Z., Yang X., Yeung K.W.K., Pan H., Wang X., Chu P.K., Wu S. (2017). Photo-inspired antibacterial activity and wound healing acceleration by hydrogel embedded with Ag/Ag@AgCl/ZnO nanostructures. ACS Nano.

[55] Han D., Li Y., Liu X., Yeung K.W.K., Zheng Y., Cui Z., Liang Y., Li Z., Zhu S., Wang X., Wu S. (2021). Photothermy-strengthened photocatalytic activity of polydopamine-modified metal-organic frameworks for rapid therapy of bacteria-infected wounds. J. Mater. Sci. Technol..

[56] Liang Y., Zhao X., Hu T., Han Y., Guo B. (2019). Mussel-inspired, antibacterial, conductive, antioxidant, injectable composite hydrogel wound dressing to promote the regeneration of infected skin. J. Colloid Interface Sci..

[57] Liang Y., Zhao X., Hu T., Chen B., Yin Z., Ma P.X., Guo B. (2019). Adhesive hemostatic conducting injectable composite hydrogels with sustained drug release and photothermal antibacterial activity to promote full‐thickness skin regeneration during wound healing. Small.

[58] Panzella L., Gentile G., D'Errico G., Della Vecchia N.F., Errico M.E., Napolitano A., Carfagna C., d'Ischia M. (2013). Atypical structural and π-electron features of a melanin polymer that lead to superior free-radical-scavenging properties. Angew. Chem. Int. Ed..

[59] Liu Y., Ai K., Ji X., Askhatova D., Du R., Lu L., Shi J. (2017). Comprehensive insights into the multi-antioxidative mechanisms of melanin nanoparticles and their application to protect brain from injury in ischemic stroke. J. Am. Chem. Soc..

[60] Mishra K., Ojha H., Chaudhury N.K. (2012). Estimation of antiradical properties of antioxidants using DPPH assay: a critical review and results. Food Chem..

[61] Selvaraj S., Fathima N.N. (2017). Fenugreek incorporated silk fibroin nanofibers a potential antioxidant scaffold for enhanced wound healing. ACS Appl. Mater. Interfaces.

[62] Wu K., Wu X., Guo J., Jiao Y., Zhou C. (2021). Facile polyphenol-europium assembly enabled functional poly (l-Lactic acid) nanofiber mats with enhanced antioxidation and angiogenesis for accelerated wound healing. Adv. Healthc. Mater..

[63] Huang X., Li L., Lyu G., Shen B., Han Y., Shi J., Teng J., Feng L., Si S., Wu J. (2018). Chitosan-coated cerium oxide nanocubes accelerate cutaneous wound healing by curtailing persistent inflammation. Inorg. Chem. Front..

[64] Ninan N., Forget A., Shastri V.P., Voelcker N.H., Blencowe A. (2016). Antibacterial and anti-inflammatory pH-responsive tannic acid-carboxylated agarose composite hydrogels for wound healing. ACS Appl. Mater. Interfaces.

[65] Zheng Y., Liang Y., Zhang D., Sun X., Liang L., Li J., Liu Y. (2018). Gelatin-based hydrogels blended with gellan as an injectable wound dressing. ACS Omega.

[66] Zhao X., Liu L., An T., Xian M., Luckanagul J.A., Su Z., Lin Y., Wang Q. (2020). A hydrogen sulfide-releasing alginate dressing for effective wound healing. Acta Biomater..

[67] He X., Liu X., Yang J., Du H., Chai N., Sha Z., Geng M., Zhou X., He C. (2020). Tannic acid-reinforced methacrylated chitosan/methacrylated silk fibroin hydrogels with multifunctionality for accelerating wound healing. Carbohydr. Polym..

[68] Mao L., Wang L., Zhang M., Ullah M.W., Liu L., Zhao W., Li Y., Ahmed A.A.Q., Cheng H., Shi Z. (2021). In situ synthesized selenium nanoparticles‐decorated bacterial cellulose/gelatin hydrogel with enhanced antibacterial, antioxidant, and anti-inflammatory capabilities for facilitating skin wound healing. Adv. Healthc. Mater..

[69] Zhang Z., Li W., Liu Y., Yang Z., Ma L., Zhuang H., Wang E., Wu C., Huan Z., Guo F. (2021). Design of a biofluid-absorbing bioactive sandwich-structured Zn-Si bioceramic composite wound dressing for hair follicle regeneration and skin burn wound healing. Bioact. Mater..

[70] Liu L., Ding Z., Yang Y., Zhang Z., Lu Q., Kaplan D.L. (2021). Asiaticoside-laden silk nanofiber hydrogels to regulate inflammation and angiogenesis for scarless skin regeneration. Biomater. Sci..

[71] Jara C.P., Wang O., Prado T.P., Ismailc A., Frank M.F., Li H., Vellosob L.A., Carlsonfg M.A., Burgessc W., Lei Y., Velanderc W.H., Araújoab E.P. (2020). Novel fibrin-fibronectin matrix accelerates mice skin wound healing. Bioact. Mater..

[72] Korntner S., Lehner C., Gehwolf R., Wagner A., Grütz M., Kunkel N., Tempfer H., Traweger A. (2019). Limiting angiogenesis to modulate scar formation. Adv. Drug Deliv. Rev..

[73] Manuel J.A., Gawronska-Kozak B. (2006). Matrix metalloproteinase 9 (MMP-9) is upregulated during scarless wound healing in athymic nude mice. Matrix Biol..

[74] Liu B., Wang Y., Miao Y., Zhang X., Fan Z., Singh G., Zhang X., Xu K., Li B., Hu Z. (2018). Hydrogen bonds autonomously powered gelatin methacrylate hydrogels with super-elasticity, self-heal and underwater self-adhesion for sutureless skin and stomach surgery and E-skin. Biomaterials.

[75] Kim M.H., Lee J., Lee J.N., Lee H., Park W.H. (2021). Mussel-inspired poly (γ-glutamic acid)/nanosilicate composite hydrogels with enhanced mechanical properties, tissue adhesive properties, and skin tissue regeneration. Acta Biomater..

[76] Bai S., Zhang X., Cai P., Huang X., Huang Y., Liu R., Zhang M., Song J., Chen X., Yang H. (2019). A silk-based sealant with tough adhesion for instant hemostasis of bleeding tissues. Nanoscale. Horiz.

[77] Lee J.S., Cho J.H., An S., Shin J., Choi S., Jeon E.J., Cho S.W. (2019). In situ self-cross-linkable, long-term stable hyaluronic acid filler by gallol autoxidation for tissue augmentation and wrinkle correction. Chem. Mater..

