# Supplementary material for: Anti-oxidant anti-inflammatory and antibacterial tannin-crosslinked citrate-based mussel-inspired bioadhesives facilitate scarless wound healing
Source: Bioact Mater. 2022 May 21;20:93–110. doi: 10.1016/j.bioactmat.2022.05.017 (PMC9131258; doi:10.1016/j.bioactmat.2022.05.017)
Supplement: Multimedia component 1 [file mmc1.docx]

Supporting Information

**Anti-oxidant anti-inflammatory and antibacterial tannin-crosslinked citrate-based mussel-inspired bioadhesives facilitate scarless wound healing**

Keke Wu^a^, Meimei Fu^a^, Yitao Zhao^a^, Ethan Gerhard^b^, Yue Li^a^, Jian Yang^b^, Jinshan Guo^a,^*

^a^Department of Histology and Embryology, NMPA Key Laboratory for Safety Evaluation of Cosmetics, School of Basic Medical Sciences, Guangdong Provincial Key Laboratory of Bone and Joint Degeneration Diseases, The Third Affiliated Hospital of Southern Medical University, Southern Medical University, Guangzhou, China

^b^Department of Biomedical Engineering, Pennsylvania State University, University Park, PA 16802, USA

*To whom correspondence should be addressed:

E-mail: [jsguo4127@smu.edu.cn](mailto:jsguo4127@smu.edu.cn) (J. Guo), Tel: +86-20-61648222.

**Table S1.** Mechanical properties of 3A-TCMBAs at dry and swollen (wet or hydration) states.

| Sample | Tensile strength (kPa) | | Elongation at break (%) | | Young’s modulus (kPa) | |
| --- | --- | --- | --- | --- | --- | --- |
|  | Dry | Swollen | Dry | Swollen | Dry | Swollen |
| 3A-TC_5%_ | 634.0 ± 72.1 | 59.9 ± 12.4 | 2367.6 ± 89.3 | 1682.1 ± 138.7 | 576 ± 76.6 | 5.5 ± 0.6 |
| 3A-TC_10%_ | 898.8 ± 92.4 | 91.2 ± 6.5 | 1988.6 ± 54.1 | 1412.9 ± 54.4 | 764.6 ± 107.2 | 20.0 ± 4.3 |
| 3A-TC_15%_ | 1081.8 ± 85.3 | 124.9 ± 4.9 | 1454.0 ± 126.7 | 1086.5 ± 50.5 | 1129.2 ± 147.7 | 39.2 ± 6.1 |


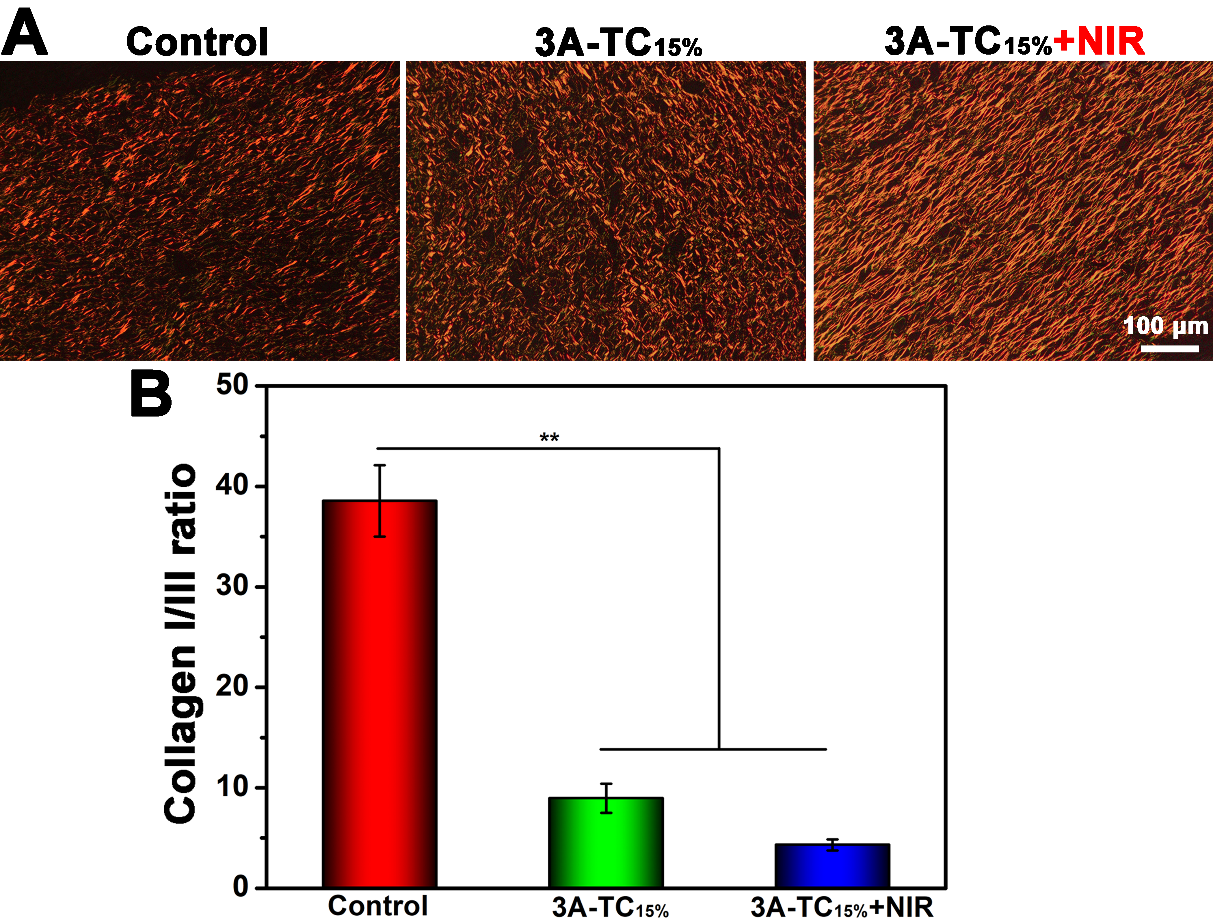


**Figure S1.** (A) Picrosirius red staining images and (B) collagen I/III ratios of wounds treated by different samples on the 21^st^ day. (***p* < 0.01)
